# Outpatient treatment and clinical outcomes of bacteriuria in veterans: A retrospective cohort analysis

**DOI:** 10.1017/ash.2022.285

**Published:** 2022-10-12

**Authors:** Suzette A. Rovelsky, Michelle Vu, Alexis K. Barrett, Kenneth Bukowski, Xiangming Wei, Muriel Burk, Makoto Jones, Kelly Echevarria, Katie J Suda, Francesca Cunningham, Karl J Madaras-Kelly, for the UTI Management Improvement Group

**Affiliations:** 1 Pharmacy Service, Boise Veterans’ Affairs Medical Center, Boise, Idaho; 2 Pharmacy Service, White River Veterans’ Affairs Medical Center, White River Junction, Vermont; 3 Center for Medication Safety (VA MedSAFE), Hines Veterans’ Affairs Medical Center, Chicago, Illinois; 4 Optum Life Sciences-HEOR, Eden Prairie, Minnesota; 5 George E. Wahlen Medical Center, Salt Lake City, Utah; 6 Veterans’ Affairs Pharmacy Benefits Management, Hines Veterans’ Affairs Medical Center, Chicago, Illinois; 7 Pittsburgh Veterans’ Affairs Medical Center, Pittsburgh, Pennsylvania; 8 Department of Medicine, University of Pittsburgh, Pittsburgh, Pennsylvania; 9 College of Pharmacy, Idaho State University, Meridian, Idaho

## Abstract

**Objective:**

To conduct a contemporary detailed assessment of outpatient antibiotic prescribing and outcomes for positive urine cultures in a mixed-sex cohort.

**Design:**

Multicenter retrospective cohort review.

**Setting:**

The study was conducted using data from 31 Veterans’ Affairs medical centers.

**Patients:**

Outpatient adults with positive urine cultures.

**Methods:**

From 2016 to 2019, data were extracted through a nationwide database and manual chart review. Positive urine cultures were reviewed at the chart, clinician, and aggregate levels. Cases were classified as cystitis, pyelonephritis, or asymptomatic bacteriuria (ASB) based upon documented signs and symptoms. Preferred therapy definitions were applied for subdiagnoses: ASB (no antibiotics), cystitis (trimethoprim-sulfamethoxazole, nitrofurantoin, β-lactams), and pyelonephritis (trimethoprim-sulfamethoxazole, fluoroquinolone). Outcomes included 30-day clinical failure or hospitalization. Odds ratios for outcomes between treatments were estimated using logistic regression.

**Results:**

Of 3,255 cases reviewed, ASB was identified in 1,628 cases (50%), cystitis was identified in 1,156 cases (36%), and pyelonephritis was identified in 471 cases (15%). Of all 2,831 cases, 1,298 (46%) received preferred therapy selection and duration for cases where it could be defined. The most common antibiotic class prescribed was a fluoroquinolone (34%). Patients prescribed preferred therapy had lower odds of clinical failure: preferred (8%) versus nonpreferred (10%) (unadjusted OR, 0.74; 95% confidence interval [CI], 0.58–0.95; *P* = .018). They also had lower odds of 30-day hospitalization: preferred therapy (3%) versus nonpreferred therapy (5%) (unadjusted OR, 0.55; 95% CI, 0.37–0.81; *P* = .002). Odds of clinical treatment failure or hospitalization was higher for β-lactams relative to ciprofloxacin (unadjusted OR, 1.89; 95% CI, 1.23–2.90; *P* = .002).

**Conclusions:**

Clinicians prescribed preferred therapy 46% of the time. Those prescribed preferred therapy had lower odds of clinical failure and of being hospitalized.

Urinary tract infection (UTI) is a common diagnosis for which antibiotics are prescribed.^1^ Increasing antibiotic resistance has made conservation of treatment options a priority.^2,3^ Optimizing antibiotic use through diagnostic and antimicrobial stewardship will help curtail the development of antibiotic resistance.

The Veterans’ Healthcare Administration (VA) has developed robust inpatient antimicrobial stewardship programs.^4^ Recent efforts have focused on improving stewardship in outpatient settings, where 75% of antibiotics are prescribed.^5,6^ The Infectious Diseases Society of America (IDSA) guidelines for UTI management in female patients are undergoing revision, and minimal guidance is available for treating UTIs in male patients.^7–10^ With calls to reduce fluoroquinolone prescriptions for cystitis, recent literature indicates changes in the prescription of antibiotic classes for uncomplicated UTI treatment.^7,9–14^ Furthermore, unnecessary antibiotic treatment of asymptomatic bacteriuria (ASB) is common, but it is hard to identify without chart review.^15^

A systematic, criteria-based quality improvement analysis of UTI management in VA outpatients was conducted by the VA Antimicrobial Stewardship Task Force (ASTF) and VA MedSAFE.^4^ To facilitate quality improvement, the primary objectives of this analysis included (1) comparison of clinical outcomes and safety events for patients treated with preferred and non-preferred therapy of ASB and UTI and (2) comparison of provider-level, database-derived measures of UTI management and their association with chart-level indicators of preferred management.

## Methods

A retrospective cohort of outpatients with positive urine cultures with monomicrobial growth (≥10^5^ CFU/mL) collected during fiscal years 2016–2019 was created. Polymicrobial cultures were excluded to reduce misinterpretation of infection versus contamination.^16^ Data were obtained from the Corporate Data Warehouse (CDW). A manual electronic health record (EHR) review was conducted by local antimicrobial stewards within VA medical centers (VAMCs) from November 2019 to April 2020. Completion of data abstraction was facilitated by monthly webinar calls with VA MedSAFE.

The cohort was constructed by randomly selecting 5–10 outpatient clinicians from each VAMC who ordered ≥15 urine cultures. Urine cultures ordered by those clinicians were randomly selected for further review. Each urine culture that met the criteria was classified as a case. Clinicians were selected from emergency departments, primary care, or specialty clinics (ie, geriatric, women’s health, or home-based primary care). The following exclusions were applied: complicated cases (eg, prostatitis, prior hospitalization within 7 days, spinal cord injury, hospital admission within 24 hours), urine cultures ordered by urology, urine cultures ordered from locations without on-site pharmacies, or urine cultures ordered when ASB treatment was appropriate (ie, pregnancy, scheduled invasive urologic procedures) (Supplementary Material). The maximum number of records reviewed per VAMC was 200 (ie, 10 clinicians with 20 positive urine cultures). Cases that met CDW-based criteria underwent further screening at the EHR-level to ensure that all criteria were met.

Chart abstraction was conducted if cases met all inclusion and no exclusion criteria. Extracted data included patient demographics (eg, urologic comorbidities), visit characteristics, signs and symptoms, antibiotic resistance risk factors, treatment rationale, laboratory results, antibiotic selection and duration, adverse drug events (ADEs), and UTI-related outcomes. Manual EHR review was employed to retrieve documentation difficult to extract within the CDW. ADEs were assessed when a patient had a return outpatient visit within 30 days with symptoms consistent with an ADE. (Training was provided on the use of the Liverpool Causation Tool.)^17^

Additional CDW data were extracted for ordering clinicians. These data included provider-level demographics, diagnostic test-ordering patterns, bacterial susceptibilities, and select clinical outcomes (Supplementary Material).

Diagnostic subgroups of UTI were classified by chart-documented signs and symptoms.^7–10,15,17^ The diagnostic definitions were based on published guidelines and were vetted in an iterative process within an ASTF workgroup.^7–10,15,17^ Acute simple cystitis (ie, cystitis) was defined as dysuria, frequency, urgency, or suprapubic pain without flank pain, costovertebral angle (CVA) tenderness, rigors, fevers (≤37.3°C), or systemic inflammatory response syndrome (SIRS) criteria. Acute pyelonephritis (ie, pyelonephritis) was defined by ≥1 symptom of cystitis plus either flank pain, CVA tenderness, rigors, fevers (>37.3°C), or SIRS criteria. ASB was defined as a lack of documentation of any of these signs or symptoms.

Preferred therapy was classified for cases based on the initial treatment received per clinical guidelines and updated guidance^7–14,18–20^ (Supplementary Material). For cystitis, preferred therapy was defined as trimethoprim-sulfamethoxazole (TMP-SMX), β-lactams (ie, cephalosporins or aminopenicillins), or nitrofurantoin. Fluoroquinolones were defined as preferred therapy only if an allergy or contraindication was documented for other preferred therapies. For pyelonephritis, preferred therapy was defined as TMP-SMX, ciprofloxacin, or levofloxacin unless antibiotic resistance risk factors were present within the past 3 months (ie, culture of gram-negative bacteria resistant to ceftriaxone, TMP-SMX or fluoroquinolones; prescription of TMP-SMX, third- or later-generation cephalosporin, or fluoroquinolone; inpatient or skilled nursing facility stay; or travel abroad).^7,10–12,20–25^ If a patient had an antibiotic-resistance risk factor, initial oral preferred therapy choices were similar; however, an intravenous or intramuscular dose of carbapenem or aminoglycoside was required.^20–25^Any therapy to which a patient had an allergy documentation, contraindication, nonsusceptibility to the antibiotic prescribed within the prior 3 months was nonpreferred. ASB preferred therapy was defined as no antibiotic prescribed.^8^

For female patients with cystitis, the preferred treatment duration was consistent with IDSA guideline recommendations, except β-lactam duration was 7 days.^7^ For male patients with cystitis, duration was preferred if the antibiotic was prescribed for 7 days (5 days for levofloxacin).^9^ For both male and female patients with pyelonephritis, preferred therapy duration was defined as ciprofloxacin for 7 days or levofloxacin for 5 days, TMP-SMX for 14 days, and β-lactam therapy for 10–14 days.^7,10^ The preferred ASB antibiotic duration was 0 days.

Outcomes extracted through chart review included return visits within 30 days for any urinary tract–related complaint, clinical treatment failure (ie, presence of new, unresolved, or worsening UTI symptoms), and ADE. Outcomes extracted from the CDW included 30-day hospitalization and 60-day new-onset *Clostridioides difficile* infection (CDI).

Descriptive statistics were used to summarize demographics and visit data. Outcomes were compared for preferred and nonpreferred antibiotic selection and antibiotic class. Subgroup analyses using a composite outcome defined as failure (ie, clinical failure plus 30-day hospitalization) were conducted for diagnoses, treatment selection, or duration by diagnoses and sex. Unadjusted odds ratios for outcomes were estimated using logistic regression. *P* values <.05 were considered statistically significant. Linear regression was used to assess the correlation between chart review–generated measures of UTI management with CDW database–derived measures for each clinician. EHR-derived measures of UTI management included the proportion of ASB cases prescribed antibiotics and the frequency of preferred therapy prescription and duration. Database-derived measures were constructed for frequency of urinary laboratory tests ordered, *International Classification of Disease, Tenth Revision* (ICD-10)–coded UTI diagnoses, antibiotic selection, and duration (Supplementary Material).

This analysis was deemed a quality improvement study by the Edward Hines, Jr, VA Hospital Institutional Review Board.^27^ Data use agreements were signed for all VAMCs.

## Results

**Fig. 1.**
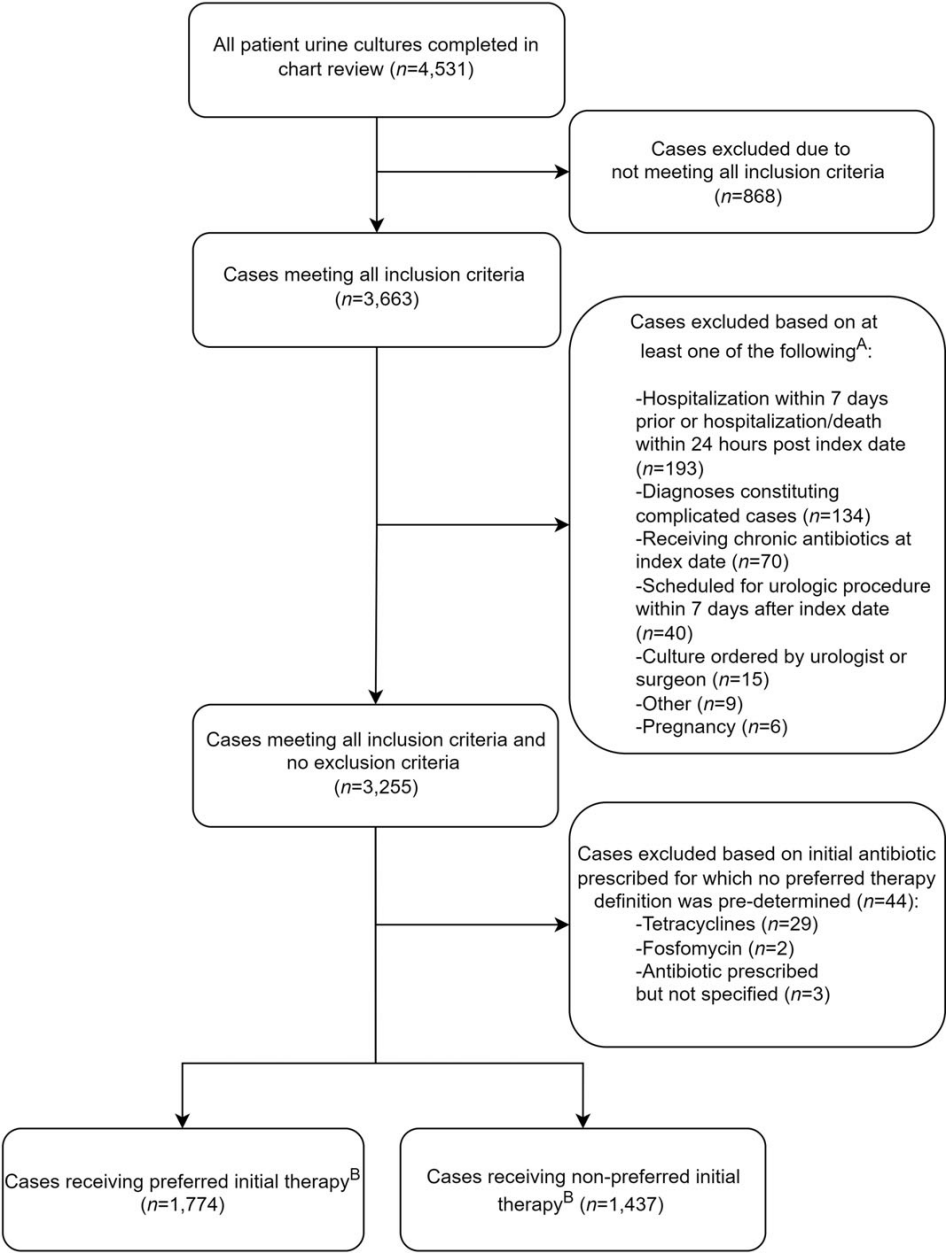
Flow diagram of outpatient UTI treatment medication utilization review. (A) Criteria are not mutually exclusive. (B) Preferred duration was further identified in 2,831 cases (eg, externally filled prescriptions with only antibiotic selection identified).

In total, 4,531 urine cultures were reviewed within 31 VAMCs. After inclusion and exclusion criteria were applied, the final cohort included 3,255 cases (Fig. 1). Common reasons for exclusion were subsequent hospitalizations or death. Overall, 259 clinicians ordered urine cultures: 191 physicians (74%) and 68 advanced-care practitioners (26%). Among them, 162 clinicians practiced in ambulatory care and 97 clinicians practiced in the emergency department. The mean patient age was 70 years (SD, ±14), and 2,490 (76%) of these 3,255 were male (Table 1). Also, 44% had ≥1 relevant comorbidity: benign prostatic hyperplasia (33%), urinary flow obstruction (8%), and nephrolithiasis (4%). Most patients were afebrile (99%). Furthermore, 12% had urinary catheterization within 7 days prior to culture collection, and 822 (25%) had no physical visit within a 6-day period (−1 to +5 days) surrounding urine-culture collection. Among these patients, 43% were prescribed an antibiotic.

**Table 1.** Characteristics of Patient Cases Meeting Utilization Review Criteria

Variable	All Cases (N=3,255)	Asymptomatic Bacteriuria (n=1,628)			Acute Simple Cystitis (n=1,156)	Uncomplicated Pyelonephritis (n=471)
		All (N=1,628)	Antibiotics (n=645)	No Antibiotics (n=983)		
Sex, male, no. (%)	2,490 (76)	1,277 (78)	494 (77)	783 (80)	860 (74)	353 (75)
Age, mean y (SD)	70 (14)	72 (14)	71 (14)	72 (13)	68 (15)	66 (14)
Temperature >37.7°C	44 (1)	0 (0)	0 (0)	0 (0)	0 (0)	44 (9)
**Patient comorbidities, no. (%)**						
≥1 comorbidity	1,428 (44)	692 (43)	274 (43)	418 (43)	542 (47)	194 (41)
BPH	1,063 (33)	515 (32)	207 (32)	308 (31)	409 (35)	139 (30)
Urinary flow obstruction	265 (8)	128 (8)	53 (8)	75 (8)	96 (8)	41 (9)
Nephrolithiasis	123 (4)	49 (3)	19 (3)	30 (3)	41 (4)	33 (7)
Dementia	108 (3)	67 (4)	30 (5)	37 (4)	36 (3)	5 (1)
**MDRO risk factors, no. (%)**						
Gram-negative antibiotic-resistant isolate within prior ≤3 mo^a^	90 (3)	41 (3)	21 (3)	20 (2)	36 (3)	13 (3)
Treatment with broad-spectrum gram-negative antibiotic within prior ≤3 mo^b^	396 (12)	178 (11)	112 (17)	66 (7)	165 (14)	53 (11)
Inpatient or SNF stay within prior ≤3 mo^c^	250 (8)	126 (8)	69 (11)	57 (6)	87 (8)	37 (9)
Travel abroad within prior ≤3 mo	9 (0.3)	0 (0)	0 (0)	0 (0)	6 (0.5)	3 (0.6)
Any MDRO risk factor	606 (19)	274 (17)	156 (24)	118 (12)	249 (22)	83 (18)
Presence of 3 positive risk factors^d^	10 (0.3)	6 (0.4)	2 (0.3)	4 (0.4)	3 (0.3)	1 (0.2)

Note. SD, standard deviation; BPH, benign prostatic hyperplasia; MDRO, multidrug-resistant organism; TMP-SMX, trimethoprim-sulfamethoxazole; SNF, skilled nursing facility.

a Antibiotic-resistant Enterobacterales included resistance to fluoroquinolone, ceftriaxone, or carbapenem cultured from any source.

b Antibiotics included fluoroquinolone, TMP-SMX, or third- or fourth-generation cephalosporin.

c Inpatient stay includes hospitalization or skilled nursing facility residence.

d Excluded travel abroad.

Moreover, ASB was identified in 1,628 cases (50%), cystitis was identified in 1,156 cases (36%), and pyelonephritis was identified in 471 cases (15%). Overall, 40% of cases with ASB were prescribed antibiotics, which was more likely if the urinalysis had pyuria [present (42%) vs absent (32%); *P* = .002], leukocyte esterase [positive (41%) vs negative (34%); *P* = .011], or microscopic hematuria [present (43%) vs absent (37%); *P* = .008].

Preferred antibiotics were selected in 1,774 (55%) of 3,211 cases (including ASB) for which preferred therapy could be defined. For cystitis and pyelonephritis, 791 (49%) of 1,600 cases received preferred antibiotic selection. Preferred duration was prescribed for 1,494 (53%) of 2,831 cases (including ASB); 1,138 (40%) of 2,831 cases received a longer duration; and 199 (7%) of 2,831 cases received a shorter duration when preferred duration could be defined (Supplementary Material). A composite of both preferred antibiotic selection and duration was identified in 1,298 (46%) of 2,831 cases.

Fluoroquinolones (34%) were the most prescribed antibiotic class. Of 312 fluoroquinolone prescriptions for cystitis, another preferred antibiotic could have been prescribed for 83%. For cases with pyelonephritis symptoms, 69 (15%) of 471 cases were treated with nitrofurantoin.

Of 471 pyelonephritis cases, 92 (20%) had at least 1 antibiotic-resistance risk factor; no case received a dose of an aminoglycoside or carbapenem. *Escherichia coli* was the most identified organism (*n* = 1,287 cases). Outpatient antibiotic susceptibilities for *E. coli* were ciprofloxacin-levofloxacin (80%), TMP-SMX (80%), nitrofurantoin (96%), ceftriaxone (94%), cefazolin (88%), amoxicillin-clavulanate (82%), gentamicin (94%), and ertapenem (100%). Additional susceptibilities are reported in the Supplementary Material.

The overall urinary symptom-related return-visit rate was 12.9% (420 of 3,255 cases). Clinical treatment failure was the predominant reason for these visits (69%). The all-cause 30-day hospitalization rate was 3.6% (117 of 3,255 cases). The overall prevalence of new-onset CDI was 0.1% (3 cases), and the documented ADE rate was 0.6% (13 of 2,028 cases).

Clinical treatment failure was lower for those who received preferred therapy than those who received nonpreferred therapy (8% vs 10%, respectively; odds ratio [OR], 0.74; 95% confidence interval [CI], 0.58–0.95; *P* = .018) (Table 2). Hospitalization rates were lower among patients who received preferred antibiotic therapies versus those who received nonpreferred antibiotic therapies (3% vs 5%, respectively; OR, 0.55; 95% CI, 0.37–0.81; *P* = .002). Female patients had a lower rate of return visits than male patients (9% vs 14%, respectively; OR, 0.64; 95% CI, 0.49–0.85; *P* = .001) and a lower rate of hospitalizations (2% vs 4%, respectively; OR, 0.37; 95% CI, 0.18–0.67; *P* < .001). There were no differences in the frequency of ADE or CDI based on therapy selection.

**Table 2.** Clinical Outcomes for Patients Who Received Preferred or Nonpreferred Outpatient UTI Treatment

Outcome	Preferred and Nonpreferred Treatment, No. (%)		Unadjusted Odds Ratio^a^ (Preferred vs Nonpreferred)		*P* Value
	Preferred Therapy (n=1,774)	Nonpreferred Therapy (n=1,437)	OR	95% CI	
Urinary-related visits within 30 days	199 (11)	211 (15)	0.73	0.59–0.91	**.003**
Presence of new, unresolved, or worsening UTI symptoms (ie, clinical failure)	138 (8)	147 (10)	0.74	0.58–0.95	**.018**
Adverse drug events	6 (0.3)	7 (0.5)	0.69	0.19–2.42	.582
Hospitalization within 30 days	46 (3)	68 (5)	0.55	0.37–0.81	**.002**

Note. OR, odds ratio; CI, confidence intervals; UTI, urinary tract infection. Bold indicates statistical significance.

a Fisher exact test.

In patients with cystitis and pyelonephritis, the composite outcome of clinical failure or hospitalization was higher for β-lactams relative to ciprofloxacin (OR, 1.89; 95% CI, 1.23–2.90; *P* = .002) and TMP-SMX (OR, 1.50; 95% CI, 1.00–2.26; *P* = .050). In particular, advanced-generation cephalosporins had a higher composite failure rate (OR, 2.82; 95% CI, 1.63–4.85; *P* < .001) relative to ciprofloxacin. Composite failure was also higher for nitrofurantoin (OR, 1.57; 95% CI, 1.01–2.44; *P* = .040) relative to ciprofloxacin (Table 3).

**Table 3.** Clinical Outcomes for Patient Cases with Acute Cystitis and Pyelonephritis by Antibiotic Class

Antibiotic (N=1,379)	Total No.	Urinary-Related Return Visit within 30 d	Clinical Failure within 30 d^a^	All-Cause Hospitalizations within 30 d	Composite Outcome (30-d Clinical Failure and All-Cause Hospitalizations)		
		No. (%)	No. (%)	No. (%)	OR	95% CI	*P* Value
Ciprofloxacin	386	61 (16)	42 (11)	9 (2)	Reference^b^		
Levofloxacin	90	12 (13)	11 (12)	6 (7)	1.53	0.78–2.89	.181
TMP-SMX	302	52 (17)	35 (12)	16 (5)	1.33	0.86–2.08	.195
β-lactams	282	61 (22)	42 (15)	21 (7)	1.89	1.23–2.90	**.002**
Amoxicillin-clavulanate	74	19 (26)	12 (16)	0 (0)	1.27	0.64–2.52	.492
First-generation cephalosporins^c^	77	9 (12)	6 (8)	7 (9)	1.33	0.63–2.67	.372
Advanced-generation cephalosporins^d^	103	26 (25)	19 (18)	12 (12)	2.82	1.63–4.85	**<.001**
Nitrofurantoin	275	54 (20)	40 (15)	13 (5)	1.57	1.01–2.44	**.040**
Other^e^	44	7 (16)	5 (11)	1 (2)	1.04	0.34–2.65	1.000

Note. OR, odds ratio; CI, confidence interval; TMP-SMX, trimethoprim-sulfamethoxazole. Bold indicates statistical significance.

a Clinical treatment failure is defined as a return visit with new, unresolved, or worsening UTI symptoms.

b Ciprofloxacin was the reference group for all individual antibiotics and antibiotic classes.

c First-generation cephalosporins included cephalexin.

d Advanced generation cephalosporins included cefuroxime, cefdinir, cefpodoxime, cefaclor, cefditoren.

e Other included doxycycline, fosfomycin, moxifloxacin, tetracycline, and unspecified.

Sex differences by drug class were also observed: female patients were more likely to have treatment failure with β-lactams (25%) than ciprofloxacin (4%) or TMP-SMX (9%): β-lactams versus ciprofloxacin (*P* = .003) and β-lactams versus TMP-SMX (*P* = .014). Male patients experienced higher composite treatment failure with β-lactams (23%) compared to ciprofloxacin (15%) but not TMP-SMX (21%): β-lactams versus ciprofloxacin (*P* = .022) and β-lactams versus TMP-SMX (*P* = .530) (Supplementary Material). Antibiotic treatment of ASB exhibited higher composite treatment failure than withholding antibiotics (12% vs 6%, respectively; OR, 2.21; 95% CI, 1.51–3.24; *P* < .001) (Supplementary Material).

For cystitis and pyelonephritis, longer duration compared to preferred duration was associated with a lower composite outcome rate (13% vs 19%, respectively; OR, 0.66; 95% CI, 0.46–0.93; *P* = .017). There was no difference in composite treatment failure based on duration of therapy for female patients; however, in male patients a longer duration compared to preferred duration was associated with a lower composite failure rate (15% vs 21%, respectively; OR, 0.66; 95% CI, 0.44–0.98; *P* = .031). The decreased composite failure rate associated with a longer duration of therapy relative to preferred duration was observed exclusively in male patients with cystitis treated with ciprofloxacin (6% vs 23%, respectively; OR, 0.22; 95% CI, 0.07–0.88; *P* = .001) (Supplementary Material).

We conducted 14 statistically significant regression analyses that compared clinician-level EHR-generated measures of UTI management and database-derived measures (Supplementary Material). No measures exhibited a strong correlation (ie, R^2^ ≥ 0.75) with clinical end points of interest.^28^ We identified 4 moderately positive (ie, R^2^ ≥0.25 to <0.75) correlations of database-derived measures with ASB treatment: (1) proportion of urinalyses ordered with antibiotics prescribed (R^2^ = 0.30; *P* < .001); (2) proportion of urine cultures ordered with antibiotics prescribed (R^2^ = 0.31; *P* < .001); (3) proportion of UTI diagnostic codes (excluding pyelonephritis) per 100 visits (R^2^ = 0.27; *P* < .001); and (4) UTI diagnostic codes (excluding pyelonephritis) with antibiotics prescribed per 100 visits (R^2^ = 0.27; *P* < .001) (Supplementary Material). A database-derived measure demonstrating moderate correlation with clinician prescribing of fluoroquinolones for cystitis was the proportion of UTI diagnostic codes (excluding pyelonephritis) treated with a fluoroquinolone (R^2^ = 0.27; *P* < .001).

## Discussion

In this retrospective cohort of outpatients with positive urine culture, half had no documented UTI-related signs or symptoms. Of these, 40% were treated with antibiotics. Patients with ASB treated with antibiotics returned more frequently than those not treated. Overall, 46% of the cohort received preferred treatment selection and duration based on historical guidelines and contemporary tertiary references. Despite cautions against prescribing fluoroquinolones for cystitis, ciprofloxacin was the most prescribed antibiotic. However, symptomatic cases prescribed β-lactams experienced UTI-related return visits and hospitalization more frequently than those treated with ciprofloxacin. Treatment failure with nitrofurantoin was also higher than with ciprofloxacin; however, a quarter of these cases had symptoms of pyelonephritis, suggesting poor identification of patients for whom nitrofurantoin is indicated. Antibiotic-resistance risk factors were identified in 20% of the pyelonephritis cases, and empirical broad-spectrum intravenous or intramuscular therapy was not initiated in any patient. Finally, no database-derived measure exhibited strong correlation with provider-level treatment of ASB or overprescribing of fluoroquinolones, but several measures demonstrated moderate correlation. Refinement of these measures may guide development of metrics to track quality improvement for UTI management over time.

The strengths of this study include the standardized data collection protocol with detailed diagnostic and treatment definitions, the ability to confirm database findings with EHR-level review, and inclusion of a relatively high proportion (24%) of female veterans, which allowed evaluation of both sexes. The premise for the cystitis preferred therapy definition was based on a fluoroquinolone-sparing approach with preference given to TMP-SMX, nitrofurantoin, or β-lactams. EHR abstraction was critical to identifying (1) patient exclusion criteria not otherwise obtainable, (2) signs and symptoms of UTI, and (3) undesirable UTI management end points.

This study also had several limitations. We relied on EHR documentation to identify the anatomical source of infection, which could have contributed to classification bias. Some cases classified as having ASB could have had UTIs; however, those with documented ASB and treatment had higher return rates than those who did not receive treatment. These findings suggest that alternative noninfectious conditions may have been misdiagnosed and undertreated. A 2-tiered approach to exclude prostatitis was used via diagnostic coding and EHR-level review, but it is possible that some cases had undocumented prostatitis. The culture time frame did not fit every clinical scenario; some cases may have had antibiotics prescribed outside the 6-day window. Although is important to recognize that the population consisted of predominantly older veterans when generalizing the findings, patients with early subsequent hospitalization and mortality were excluded. This analysis was conducted as a quality improvement project, and the outcome analyses were not risk-adjusted but stratified, which limits causal interpretations. Assessment of the association between index culture antibiotic sensitivity and treatment outcome is beyond the scope of our quality improvement project. To minimize the burden on chart abstractors, susceptibility data were extracted from the CDW. Only 17% of patients with cystitis or pyelonephritis treated with antibiotics had a reported sensitivity interpretation to the prescribed antibiotic. These findings are likely due to individual facility variability on sensitivity reporting (ie, manual EHR entry). In cases for whom sensitivities could be interpreted, only 2.3% of these cases were resistant to a preferred therapy. A sensitivity analysis was conducted by removing discordant treatment-susceptibility cases, which did not alter the statistical significance of the findings. Finally, although regression analyses were limited to clinicians with ≥15 urine cultures, the accuracy to identify metrics focusing specifically on clinician-level management of ASB or cystitis was limited.

Our findings support recommendations to withhold ASB treatment in cases without indications for prophylaxis, and prudent assessment of symptoms is essential to appropriate treatment.^9^ Compared to a similar VHA inpatient UTI analysis conducted in 2013–2014, ASB was seen almost as frequently in the outpatient setting but was treated less often than in the inpatient setting.^15^ Clinician awareness and outpatient antibiotic stewardship may have led to reduced ASB treatment over time; however, minimal change in outpatient ASB treatment by cohort year (43% in 2016 vs 38% in 2019) was observed. US prescribing data suggest that fluoroquinolone use has decreased by half between 2015 and 2019.^28^ However, our data suggest that in contrast to FDA warnings, clinicians still frequently prescribe fluoroquinolone antibiotics for cystitis. We also identified a significant number of cases of oral antibiotics with reduced systemic concentrations (ie, nitrofurantoin, β-lactams) prescribed for pyelonephritis when fluoroquinolones may have been preferred. Contemporary tertiary references suggest that men with cystitis and an absence of prostatitis symptoms may be treated with 7 days of antibiotics.^9^ A recent randomized controlled noninferiority trial of TMP-SMX and ciprofloxacin for afebrile UTI in men with limited comorbid complications found no difference in clinical failure rates between 7 and 14 days of therapy; further analysis of the preferred duration in male patients is needed.^9,29,30^ The transition away from fluoroquinolone to non–fluoroquinolone-based UTI treatment, especially toward β-lactams, has been well documented.^13,14,31^ However, sufficiently powered clinical efficacy studies are lacking in many patient populations for β-lactams; particularly for advanced-generation cephalosporins. Some studies have found diminished efficacy of advanced-generation cephalosporins relative to ciprofloxacin in women.^32^

Recent studies have identified antibiotic-resistance risk factors for UTIs, and well-established references recommend utilizing broad-spectrum coverage with a carbapenem or aminoglycoside in addition to oral therapy for these cases.^9,10,20–25^ Utilizing parenteral antibiotics may prove necessary in areas with reduced TMP-SMX and fluoroquinolone susceptibilities. Further validation of treatment outcomes based on these risk factors is needed.

Future work includes development of an interventional strategy to mitigate the findings. Academic detailing campaigns have been successful at improving management of other infectious syndrome-based conditions within the VHA, and a clinician-directed academic detailing campaign for UTIs is under development.^33^ Additional work is needed to develop and validate EHR-based metrics to capture UTI diagnosis and management, which may facilitate continuous, systematic quality review. With the heterogeneity of genitourinary tract presentations, an aging population, and the large shift in treatment away from fluoroquinolone treatment, research is needed to determine the role of β-lactam treatment for UTIs. Finally, incorporation and validation of antibiotic risk-factor identification into treatment algorithms and their impact on clinical outcomes is needed.

In conclusion, this analysis demonstrated that the preferred treatment in a cohort of veterans with positive urine cultures was prescribed less than half the time. Those who received preferred therapy had a lower odds of clinical failure and being hospitalized with no significant impact on adverse events. Clinicians should carefully differentiate upper- and lower-tract UTI symptoms to aid in selection of preferred therapy. Clinicians should also assess when to use specific antimicrobials, including the use of fluoroquinolones, on a case-by-case basis. These findings suggest clinicians could benefit from educational and interventional strategies that target appropriate selection and duration of therapy.

## Supplementary material

For supplementary material accompanying this paper visit https://doi.org/10.1017/ash.2022.285. click here to view supplementary material

## Research transparency and reproducibility

Data have been retained in accordance with VA and VHA policy including the following: VA Handbook 5011/5, Chapter 4 (Alternative Workplace Arrangements); VA Directive and Handbook 6500, Information Security Program; VA Directive and Handbook 6502, Privacy Program; VHA Directive 1605 Privacy Program, VHA Handbook 1605.1 and 1605.2 and VHA Handbook 1200.12, Use of Data and Data Repositories in VHA Research. None of the VHA data, any data extracted or derived from this report, or other data files provided by the VHA, can be released to any other organization or individual external to your organization without the appropriate approval of the transferring VHA office.

## References

[1] Simmering JE , Tang F , Cavanaugh, et al. The increase in hospitalizations for urinary tract infections and the associated costs in the United States, 1998–2011. Open Forum Infect Dis 2017;4:ofw281. 28480273 10.1093/ofid/ofw281 PMC5414046

[2] Antibiotic resistance threats in the United States, 2019. Centers for Disease Control and Prevention website. https://www.cdc.gov/drugresistance/pdf/threats-report/2019-ar-threats-report-508.pdf. Published 2019. Accessed September 19, 2022.

[3] Healthy people 2030. US Department of Health and Human Services Office of Disease Prevention and Health Promotion website. https://health.gov/healthypeople. Published August 2020. Accessed August 20, 2021.

[4] Kelly AA , Jones MM , Echevarria KL , et al. A report of the efforts of the Veterans’ Health Administration National Antimicrobial Stewardship Initiative. Infect Control Hosp Epidemiol 2017;38:513–520. 28118861 10.1017/ice.2016.328

[5] Suda KJ , Hicks LA , Roberts RM , et al. Antibiotic expenditures by medication, class, and healthcare setting in the United States, 2010–2015. Clin Infect Dis 2018;66:185–190. 29020276 10.1093/cid/cix773 PMC9454312

[6] Sanchez GV , Fleming-Dutra KE , Roberts RM , et al. Core elements of outpatient antibiotic stewardship. MMWR Recomm Rep 2016;65(6):1–12. 10.15585/mmwr.rr6506a1 27832047

[7] Gupta K , Hooton TM , Naber KG , et al. International clinical practice guidelines for the treatment of acute uncomplicated cystitis and pyelonephritis in women: a 2010 update by the Infectious Diseases Society of America and the European Society for Microbiology and Infectious Diseases. Clin Infect Dis 2011;52:e103. 21292654 10.1093/cid/ciq257

[8] Nicolle LE , Gupta K , Bradley SF , et al. Clinical practice guideline for the management of asymptomatic bacteriuria: 2019 update by the Infectious Diseases Society of America. Clin Infect Dis 2019;68:1611–1615. 31506700 10.1093/cid/ciz021

[9] Hooton TM. Acute simple cystitis in men. UpToDate website. https://www.uptodate.com/contents/acute-simple-cystitis-in-men. Updated July 20, 2020. Accessed June 22, 2021.

[10] Hooton TM , Gupta K. Acute complicated urinary tract infection (including pyelonephritis) in adults. UpToDate website. https://www.uptodate.com/contents/acute-complicated-urinary-tract-infection-including-pyelonephritis-in-adults. Updated March 19, 2021. Accessed June 22, 2021.

[11] Fosse PE , Brinkman KM , Brink HM , Conner CE , Aden JK , Giancola SE. Comparing outcomes among outpatients treated for pyelonephritis with oral cephalosporins versus first-line agents. Int J Antimicrob Agents 2022;59:106560. 35259485 10.1016/j.ijantimicag.2022.106560

[12] Fox MT , Melia MT , Same RG , Conley AT , Tamma PD. A seven-day course of TMP-SMX may be as effective as a seven-day course of ciprofloxacin for the treatment of pyelonephritis. Am J Med 2017;130:842–845. 28216442 10.1016/j.amjmed.2017.01.025 PMC5632565

[13] FDA Drug Safety Communication: FDA updates warnings for oral and injectable fluoroquinolone antibiotics due to disabling side effects. US Food and Drug Administration website. https://www.fda.gov/drugs/drug-safety-and-availability/fda-drug-safety-communication-fda-updates-warnings-oral-and-injectable-fluoroquinolone-antibiotics. Updated December 20, 2018. Accessed June 21, 2021.

[14] Kulchavenya E. The best rules for antimicrobial stewardship in urogenital tract infections. Curr Opin Urol 2020;30:838–844. 32881727 10.1097/MOU.0000000000000817

[15] Spivak ES , Burk M , Zhang R , et al. Management of bacteriuria in Veterans’ Affairs hospitals. Clin Infect Dis 2017;65:910–917. 28531289 10.1093/cid/cix474

[16] Xu R , Deebel N , Casals R , et al. A new gold rush: a review of current and developing diagnostic tools for urinary tract infections. Diagnostics 2021;11:479. 33803202 10.3390/diagnostics11030479 PMC7998255

[17] Gallagher RM , Kirkham JJ , Mason JR , et al. Development and interrater reliability of the Liverpool adverse drug reaction causality assessment tool. PLoS One 2011;6:e28096. 22194808 10.1371/journal.pone.0028096 PMC3237416

[18] Rubin RH , Shapiro ED , Vincent TA , et al. Evaluation of new anti-infective drugs for the treatment of urinary tract infection. Clin Infect Dis 1992;15:S216–S227. 1477233 10.1093/clind/15.supplement_1.s216

[19] Piraux A , Faure S , Naber KG , Alidjanov JF , Ramond-Roquin A. Changes in the management of urinary tract infections in women: impact of the new recommendations on antibiotic prescribing behavior in France, between 2014 and 2019. BMC Health Serv Res 2021;21:612. 34182991 10.1186/s12913-021-06653-4 PMC8240268

[20] Langner JL , Chiang KF , Stafford RS. Current prescribing practices and guideline concordance for the treatment of uncomplicated urinary tract infections in women. Am J Obstet Gynecol 2021;225:272.e1–272.e11. 10.1016/j.ajog.2021.04.218 33848538

[21] Talan DA , Takhar SS , Krishnadasan A , et al. Emergence of extended-spectrum β-lactamase urinary tract infections among hospitalized emergency department patients in the United States. Ann Emerg Med 2021;77:32–43. 33131912 10.1016/j.annemergmed.2020.08.022

[22] Linsenmeyer K , Strymish J , Gupta K. Two simple rules for improving the accuracy of empiric treatment of multidrug-resistant urinary tract infections. Antimicrob Agents Chemother 2015;59:7593–7596. 26416859 10.1128/AAC.01638-15 PMC4649203

[23] Gupta K , Bhadelia N. Management of urinary tract infections from multidrug-resistant organisms. Infect Dis Clin North Am 2014;28:49–59. 24484574 10.1016/j.idc.2013.10.002

[24] Foxman B. Editorial commentary: extended-spectrum β-lactamase–producing *Escherichia coli* in the United States: time to rethink empirical treatment for suspected *E. coli* infections? Clin Infect Dis 2013;56:649–651. 23150212 10.1093/cid/cis947

[25] Johnson L , Sabel A , Burman WJ , et al. Emergence of fluoroquinolone resistance in outpatient urinary *Escherichia coli* isolates. Am J Med 2008;121:876–884. 18823859 10.1016/j.amjmed.2008.04.039

[26] Colgan R , Johnson JR , Kuskowski M , Gupta K. Risk factors for trimethoprim-sulfamethoxazole resistance in patients with acute uncomplicated cystitis. Antimicrob Agents Chemother 2008;52:846–851. 18086847 10.1128/AAC.01200-07 PMC2258492

[27] VHA Handbook 1058.05, VHA operations activities that may constitute research. Veterans’ Health Administration website. www.research.va.gov/resources/policies/oro-120811.cfm. Accessed June 4, 2021.

[28] Moore DS , Notz WI , Flinger MA. The Basic Practice of Statistics, Sixth *Edition.* New York: WH Freeman; 2013:138.

[29] Germanos GJ , Trautner BW , Zoorob RJ , et al. No clinical benefit to treating male urinary tract infection longer than seven days: an outpatient database study. Open Forum Infect Dis 2019;6:ofz216. 31249844 10.1093/ofid/ofz216 PMC6580996

[30] Drekonja DM , Trautner B , Amundson C , Kuskowski M , Johnson JR. Effect of 7 vs 14 days of antibiotic therapy on resolution of symptoms among afebrile men with urinary tract infection: a randomized clinical trial. JAMA 2021;326:324–331. 34313686 10.1001/jama.2021.9899 PMC8317010

[31] Buehrle DJ , Wagener MM , Clancy CJ. Outpatient fluoroquinolone prescription fills in the United States, 2014 to 2020: assessing the impact of Food and Drug Administration safety warnings. Antimicrob Agents Chemother 2021;65:e0015121. 33875430 10.1128/AAC.00151-21 PMC8218674

[32] Hooton TM , Roberts PL , Stapleton AE. Cefpodoxime vs ciprofloxacin for short-course treatment of acute uncomplicated cystitis: a randomized trial. JAMA 2012;307:583–589. 22318279 10.1001/jama.2012.80 PMC3736973

[33] Madaras-Kelly K , Hostler C , Townsend M , et al. Impact of implementation of the core elements of outpatient antibiotic stewardship within Veterans’ Health Administration Emergency Department and Primary Care Clinics on Antibiotic Prescribing and Patient Outcomes. Clin Infect Dis 2021;73:e1126–e1134. 33289028 10.1093/cid/ciaa1831

