# Supplementary material for: Outpatient treatment and clinical outcomes of bacteriuria in veterans: A retrospective cohort analysis
Source: Antimicrob Steward Healthc Epidemiol. 2022 Oct 12;2(1):e168. doi: 10.1017/ash.2022.285 (PMC9726514; doi:10.1017/ash.2022.285)
Supplement: Supplementary file 1 [file S2732494X22002856sup001.docx]

Contents

[1. Data Collection Form 2](#_Toc101733617)

[2. Metric list for database (CDW) and chart review (EHR) 13](#_Toc101733618)

[3. Preferred therapy selection and duration definitions 13](#_Toc101733619)

[4. Urinary Isolate Breakdown 15](#_Toc101733620)

[5. Pathogen sensitivities 16](#_Toc101733621)

[6. Additional outcome analyses 16](#_Toc101733622)

[7. Adverse Drug Event Outcomes 21](#_Toc101733623)

[8. Linear regression Analyses of Provider-level Database-derived and Chart-derived Measure of UTI Management. 22](#_Toc101733624)

# 1. Data Collection Form

**Medication Utilization Evaluation For Management of Bacteriuria in Outpatient Setting**

The positive urine culture identified on the index date should fall within the evaluation timeframe: **October 1, 2016 – September 30, 2019**

| Reviewer Initials | VISN | Station | Date Case Report Completed | Positive Outpatient Urine culture collection date **(INDEX DATE)** | Provider identification number | Patient identification number | Sex |
| --- | --- | --- | --- | --- | --- | --- | --- |
| 🞎🞎🞎 | 🞎🞎 |  | _____/_____/____________  (mm/dd/yyyy) | _____/_____/____________  (mm/dd/yyyy) | 🞎🞎🞎🞎🞎🞎 | 🞎🞎🞎🞎🞎🞎 | - - Male   - Female |

**General Criteria:**

**1. Evaluate that inclusion criteria are met (all criteria must be met)**

- a. Positive urine culture with only one organism with a quantitative count of > 10^5^ cfu/ml for any bacterial organism.
- b. Positive urine culture ordered by a provider who is a physician, nurse practitioner, or physician assistant
- c. Positive urine culture ordered through a VA Emergency Department, Primary care, Geriatric, Women’s, or HBPC Specialty Clinic outpatient setting.

**2a. Evaluate if any exclusion criteria are met (check all that apply)**

- 1.EXCLUDE if ordering provider is a Urology or Surgery service provider.
- 2. EXCLUDE if patient was admitted to an acute care unit within 24 hours after the culture index date
- 3. EXCLUDE if patient dies within 24 hours after the culture index date.
- 4. EXCLUDE if patient had an inpatient admission within 7 days prior to culture index date.
- 5. EXCLUDE if patient had an index urine culture containing *Candida* spp., alone or in combination with any other organisms (e.g., *E*. *coli* and *Candida albicans*).

2b. **Evaluate if any exclusion criteria are met (check all that apply)**

- 1.EXCLUDE if patient underwent or was scheduled for urologic procedures that may cause mucosal trauma within 7 days after index date (check all that apply)

| - - - - a. Transurethral resection of the prostate | - - - - b. Prostate biopsy | - - - - c. Cystoscopy | - - - - d. Other (i.e. orchiectomy)   Describe: |
| --- | --- | --- | --- |

- 2.EXCLUDE if patient was receiving chronic systemic antibiotics at culture index date.
- 3.EXCLUDE- FOR FEMALES: if patient was pregnant on the culture index date
- 4.EXCLUDE if patient had ANC <500 cells/mm3 within 7 days prior to the culture index date
- 5.EXCLUDE if patient had prior Solid Organ or Bone Marrow transplantation prior to the culture index date
- 6.EXCLUDE if patient is receiving hemodialysis or peritoneal dialysis within 7 days prior to the culture index date
- 7.EXCLUDE- FOR MALES: if patient was diagnosed with Epididymitis with or without orchitis on the culture index date
- 8.EXCLUDE- FOR MALES: if the patient had a suspected /confirmed diagnosis of acute or chronic prostatitis on the culture index date
- 9.EXCLUDE if patient had a spinal cord injury resulting in paralysis prior to the culture index date.
- 10.EXCLUDE if patient had an ileal conduit or other urinary diversion post cystectomy in place on the culture index date.
- 11.EXCLUDE if patient has urinary stents or nephrostomy tubes in place or placed on the culture index date.

***** Stop here if ANY criteria in item 1 ARE NOT met, or ANY criteria in item 2 ARE met. Submit the case report form. ****

**Encounter Information**

**3. Did a physical visit happen with a provider within 2 days before or 3 days after collection of the urine culture?**

**🞏 a. Yes 🞏 b. No**

NOTE: If the provider received workload or encountered credit for the physical visit, then you would mark YES on Question 3. If the provider did NOT receive workload or encountered credit for the physical visit, then you would mark NO.

**3a1. If YES to question 3, did any visit documentation mention urinary tract evaluation for infection or collection of the urine culture? 🞏 a. Yes 🞏** b. No

**3a2. If YES to question 3, was the visit performed by the same provider that ordered the urine culture? 🞏 a. Yes 🞏** b. No

**3b1. If there was not a physical visit with the urine culture ordering provider, was there a physical visit with a different provider/healthcare professional (e.g. nurse) within 2 days before or 3 days after collection of the urine culture? 🞏 a. Yes 🞏 b. No**

3b2. If YES to question 3b1, who was the visit with?

🞏 a. Alternate primary provider

🞏 b. ED provider

🞏 c. Nurse

🞏 d. Specialty provider

🞏 e. Other: Please describe:

3b3. If NO to question 3 , was there a progress note entered within 2 days before or 3 days after the urine

culture collection that mentions a urinary tract infection or the urine culture? 🞏 a. Yes 🞏 b. No

**4a. Was a urinary tract-related suspected diagnosis documented in visit notes on the index date?**

- a. Yes 🞏 b. No

4b. If YES to 4a., select all that apply (If an ICD10 code was documented, write the code in the last column):

|  | Select all that apply | | List ICD10 code(s): |
| --- | --- | --- | --- |
| 4b1. Asymptomatic bacteriuria | 🞏 4b1a. Written in provider note | 🞏 4b1b. ICD10 code in encounter information | 4b1c. [text box] |
| 4b2. Cystitis | 🞏 4b2a. Written in provider note | 🞏 4b2b. ICD10 code in encounter information | 4b2c. [text box] |
| 4b3. Pyelonephritis | 🞏 4b3a. Written in provider note | 🞏 4b3b. ICD10 code in encounter information | 4b3c. [text box] |
| 4b4. Urinary tract infection | 🞏 4b4a. Written in provider note | 🞏 4b4b. ICD10 code in encounter information | 4b4c. [text box] |

**5. Was there another (non-UTI) diagnosis that was referenced as a reason for prescribing antibiotics documented in visit notes on the index date?**

🞏 1. Yes 🞏 2. No

**5b. If yes, select the diagnostic category (Scroll down menu (select all that apply): 1.Pneumonia, 2.COPD exacerbation, 3.Sinusitis, 4.Pharyngitis,5. Otitis, 6.SSTI, 7.GI, 8.STD, 9. other)**

**Patient’s Past Medical History:**

**6. Did patient have any of the following UTI -relevant antibiotic-resistant risk factors on culture index date? (check all that apply)**

| - a. Hospitalization or skilled nursing facility residence in past 3 months - b. Fluoroquinolone, TMP/SMX, or 3^rd^/4^th^ generation cephalosporin use in past 3 months - c. International travel within the past 3 months - d. Fluoroquinolone, ceftriaxone, or carbapenem-resistant Enterobacteriaceae isolated from any source in **prior 3** months - e. Fluoroquinolone, ceftriaxone, or carbapenem-resistant Enterobacteriaceae isolated from any source in **prior 12** months |
| --- |
| - f. None |

**7. Was the patient previously on any antibiotic therapy within the 2 weeks prior to index date? 🞏 a. Yes 🞏 b. No**

**8. Did patient have any of the following baseline characteristics on index date? (based on prior clinic or inpatient notes, check all that apply)**

| - a. Urolithiasis (current condition only) | - b. Benign prostatic hyperplasia | - c. Urinary flow obstruction   (e.g., ureteral or bladder outlet obstruction) | - d. Prostate cancer undergoing radiation/chemotherapy |
| --- | --- | --- | --- |
| - e. Neurogenic bladder | - f. Nephrolithiasis | - g. Bladder cancer | - h. Dementia (including Alzheimer) |
| - i. History of Transurethral resection of the prostate (TURP) | - j. Multiple Sclerosis | k. None |  |

**8. Urinary catherization within 1 week prior to the index date?** 1. Yes 🞏 2. No

**Urinary Tract Signs and Symptoms:**

**9. Were any of the following documented as present within 2 days before the culture index date until either (1) 3 days after -or- (2) 24 hours after first antibiotic was prescribed (whichever is first)? (check all that apply)**

| **9a. Signs and symptoms of UTI** | |  |  |
| --- | --- | --- | --- |
| 1.Dysuria | | 🞎a. Yes 🞎b. No 🞎c. Not Documented 🞎d. Provider could not assess^*^ |  |
| 2.Frequency | | 🞎a. Yes 🞎b. No 🞎c. Not Documented 🞎d. Provider could not assess^*^ |  |
| 3.Urgency | | 🞎a. Yes 🞎b. No 🞎c. Not Documented 🞎d. Provider could not assess^*^ |  |
| 4.Suprapubic pain on examination | | 🞎a. Yes 🞎b. No 🞎c. Not Documented 🞎d. Provider could not assess^*^ |  |
| 5.Acute gross (macroscopic) hematuria | | 🞎a. Yes 🞎b. No 🞎c. Not Documented 🞎d. Provider could not assess^*^ |  |
| 6.Flank pain | | 🞎a. Yes 🞎b. No 🞎c. Not Documented 🞎d. Provider could not assess^*^ |  |
| 7.Costovertebral (CVA) tenderness | | 🞎a. Yes 🞎b. No 🞎c. Not Documented 🞎d. Provider could not assess^*^ |  |
| 8.Rigors (or shaking chills) | | 🞎a. Yes 🞎b. No 🞎c. Not Documented 🞎d. Provider could not assess^*^ |  |
| 9. Subjective Fever | | 🞎a. Yes 🞎b. No 🞎c. Not Documented 🞎d. Provider could not assess^*^ |  |
|  | | |  |
|  | | |  |
| **9b.Additional Non-specific symptoms** | |  |  |
| 1.Altered mental status from baseline | | 🞎a. Yes 🞎b. No 🞎c. Not Documented 🞎d. Provider could not assess^*^ |  |
| 2.Recent Fall or Falls | | 🞎a. Yes 🞎b. No 🞎c. Not Documented 🞎d. Provider could not assess^*^ |  |
| 3.Malaise/Lethargy | | 🞎a. Yes 🞎b. No 🞎c. Not Documented 🞎d. Provider could not assess^*^ |  |
| 4.Nausea/Vomiting | | 🞎a. Yes 🞎b. No 🞎c. Not Documented 🞎d. Provider could not assess^**^ |  |
| 5.Pelvic discomfort (symptom) | | 🞎a. Yes 🞎b. No 🞎c. Not Documented 🞎d. Provider could not assess^*^ |  |
| 6.Foul smelling urine | | 🞎a. Yes 🞎b. No 🞎c. Not Documented 🞎d. Provider could not assess^*^ |  |
| 7.Cloudy or dark urine | | 🞎a. Yes 🞎b. No 🞎c. Not Documented 🞎d. Provider could not assess^*^ |  |

*^*^Example: patient was demented and was not able to verbalize symptoms. Therefore, the answer would be marked as “provider could not assess”.*

*This option should NOT be used if the provider documented the presence or absence of the relevant signs or symptoms of UTI; nor should it be used if the provider failed to assess signs or symptoms of UTI*

**10a. Did patient meet any of the following SIRS criteria on the culture index date? (check all that apply)**

| **SIRS criteria** | **On index date** |
| --- | --- |
| 1. Temperature < 36°C (96.8°F) or > 38.3°C (100.9°F) | 🞎a. Yes 🞎b. No 🞎c. Not Documented |
| 2. HR > 90 bpm | 🞎a. Yes 🞎b. No 🞎c. Not Documented |
| 3. RR > 20bpm **or** PaCO_2_ < 32mmHg | 🞎a. Yes 🞎b. No 🞎c. Not Documented |
| 4. WBC < 4,000/mm^3^ **or** > 12000/mm^3^ **or** > 10% bands | 🞎a. Yes 🞎b. No 🞎c. Not Documented |

**10b. Did the patient have a temperature > 99.9 F (37.7C) on the culture index date?**

🞏 1.Yes 🞏 2. No 🞏 3. Not documented

**11. How was the index urine culture collected? (check only one box)**

| - a. Clean catch or midstream | - b. Catheter | - c. Not Specified |
| --- | --- | --- |

**Index Urine Culture Collection**

**12. Index Culture Originating Clinic (check only one box)**

🞏 a. Primary Care Clinic 🞏 b. Urgent Care Clinic 🞏 c. Emergency Dept 🞏 d. Specialty clinics: (Drop down for (1) Women’s, (2) Geri, (3) HBPC)

🞏 e. Unknown

**13. Index Culture Ordering Provider Characteristics (check only one box)**

🞏 a. Staff Physician 🞏 b. Advanced Practice Providers (ie., PA, NP) 🞏 c. Med Resident/Fellow 🞏 d. Cannot determine

**14. Was the culture collected as a “reflex” culture? a. 🞏 Yes 🞏 b. No 🞏 c. Cannot determine**

**15. Index Culture Microbiologic Results: Please list any comments about organisms reported (note: culture should only be included if one bacterial species with >100K):** [text box here]

**16. Was a urinalysis (UA) obtained within 24 hours of index date? (check only the one that applies)**

| - a. Yes (if answer “yes”, proceed to #17) | - b. No (if answer “no”, proceed to #18) |
| --- | --- |

**17. If urinalysis was obtained within 24 hours of index date (check all that apply, enter N/A if no value is available.)**

| **Urinalysis**  **(If > 1 UAs were available, use the UA closest to the time of urine culture collection)** |
| --- |
| 🞎 a. Evidence of pyuria (> 5 WBCs/high power field ) |
| 🞎 b. Positive for leukocyte esterase |
| 🞎 c. Positive for nitrite |
| 🞎 d. Positive for hematuria (≥ 3 RBCs/high power field) |
| 🞎 e. N/A |

**Antibiotic Treatment:**

**18. Was antibiotic therapy initiated within 2 days before or 3 days after the index date? 🞏 a. Yes 🞏 b. No**

**If antibiotic therapy was initiated within 2 days or 3 days after the index date, then proceed to questions 19-23. If no, proceed to question 24.**

**19. If yes to Question 18, select the number of days before or after the index urine culture that the provider initiated the antibiotic (scroll down with -2,-1,0,1,2,3 days post culture date)**

**20a. Record the serum creatinine value obtained nearest to the date of first antibiotic prescription (scroll down with <0.4 through 2.5, > 2.5 in tenths of mg/dl.). Select N/A if none available (20b).**

Scroll down option as followed (in mg/dL):

20a1. <0.4

20a2. 0.4

20a3. 0.5

20a4. 0.6

etc. with the second to last option as “>2.5” and the last option as “N/A”

**21a. Record the patient’s weight in kg obtained nearest to the date of first antibiotic prescription. (scroll down with <50 through 120 kg , > 120 in Kg. Select N/A if none available (21b).**

21 a. would be as followed (in kg):

21 a1. <50

21 a2. 50

21 a3. 51

etc.

**22a. Record the patient’s height in inches obtained nearest to the date of first antibiotic prescription. (scroll down with 50 through 80 inches Select N/A if none available). (22b)**

22 a. would be as followed (in inches):

22 a1. <50

22 a2. 50

22 a3. 51

etc.

**23. Indicate if the patient had an antibiotic allergy listed at the time antibiotic therapy was initiated (Select all that apply):**

- NKDA (23a)
- Penicillins (23b)
- Cephalosporins (23c)
- TMP/SMX (23d)
- Nitrofurantoin (23e)
- Fluoroquinolones (23f)
- Fosfomycin (23g)
- Other systemically administered antibiotic (23h)

**24 .** For the following antibiotics, indicate the following (select one option per row):

| **a. SMX/TMP** | 🞏 1. Prescribed | 🞏 2. NOT prescribed, NO rationale provided | 🞏 3. NOT prescribed, rationale provided (go to question 25.) |
| --- | --- | --- | --- |
| **b. Nitrofurantoin** | 🞏 1. Prescribed | 🞏 2. NOT prescribed, NO rationale provided | 🞏 3. NOT prescribed, rationale provided (go to question 25.) |
| **c. Fosfomycin** | 🞏 1. Prescribed | 🞏 2. NOT prescribed, NO rationale provided | 🞏 3. NOT prescribed, rationale provided (go to question 25.) |
| **d. Beta-lactam** | 🞏 1. Prescribed | 🞏 2. NOT prescribed, NO rationale provided | 🞏 3. NOT prescribed, rationale provided (go to question 25.) |

**If option #3 was NOT selected for any of 24a-d, move on to question 26.**

**25. If option #3 WAS selected for any of 24a-d, fill out the table below (select ALL that apply):**

| **1. SMX/TMP** | 🞏 a. Kidney function | 🞏 b. Adverse drug reaction | 🞏 c. Drug-drug interaction | 🞏 d. Drug-disease interaction | 🞏 e. Allergy | 🞏 f. Antibiotic resistance noted within the last 3 months | 🞏 g. Other | 🞏 h. No rationale documented |
| --- | --- | --- | --- | --- | --- | --- | --- | --- |
| **2. Nitrofurantoin** | 🞏 a. Kidney function | 🞏 b. Adverse drug reaction | 🞏 c. Drug-drug interaction | 🞏 d. Drug-disease interaction | 🞏 e. Allergy | 🞏 f. Antibiotic resistance noted within the last 3 months | 🞏 g. Other | 🞏 h. No rationale documented |
| **3. Fosfomycin** | 🞏 a. Kidney function | 🞏 b. Adverse drug reaction | 🞏 c. Drug-drug interaction | 🞏 d. Drug-disease interaction | 🞏 e. Allergy | 🞏 f. Antibiotic resistance noted within the last 3 months | 🞏 g. Other | 🞏 h. No rationale documented |
| **4. Beta-lactam** | 🞏 a. Kidney function | 🞏 b. Adverse drug reaction | 🞏 c. Drug-drug interaction | 🞏 d. Drug-disease interaction | 🞏 e. Allergy | 🞏 f. Antibiotic resistance noted within the last 3 months | 🞏 g. Other | 🞏 h. No rationale documented |

**26. Was there a ONE-TIME dose of IV or IM antibiotics administered during the visit? 🞏 a. Yes 🞏 b. No c.🞏 N/A (no visit)**

**If yes, which antibiotic? (Select all that apply)**

1.Ceftriaxone 🞏

2.Gentamicin 🞏

3.Ertapenem/Meropenem 🞏

4.Ciprofloxacin/Levofloxacin 🞏

5.Other 🞏 Please specify

**27. Please list antibiotic(s) and their duration (exclude the one-time IM/IV dose). If there was no systemic oral antibiotic therapy initiated within 2 days before or 3 days after the index date, then proceed to question 28:**

| **A** | **B** | **C** | **D** |
| --- | --- | --- | --- |
| **Antibiotic Name (Allow for multiple antibiotics to be selected; if “Other” is selected, please provide antibiotic name)** | **Duration (# days’ supply; provide for each antibiotic prescribed)** | **Date Filled**  **(MM/DD/YY;**  **provide for each antibiotic prescribed)** | **Source of Antibiotic Filled** |
| Drop Down – 1. Amoxicillin, 2. Amox-Clav., 3.Cefaclor, 4. Cefadroxil, 5. Cefdinir, 6. Cefditoren,7. Cefixime, 8. Cefpodoxime, 9. Cefprozil, 10. Ceftibuten, 11. Cefuroxime, 12. Cephalexin,13. Ciprofloxacin,14.. Doxycycline, 15. Fosfomycin,16. Levofloxacin, 17. Linezolid, 18. Minocycline, 19. Moxifloxacin, 20. Nitrofurantoin, 21. Tetracycline, 22. TMP/SMX, 23. Antibiotic prescribed but not specified, 24. Other _______________ | ____________________(1)  🞏 Not Documented(2) | ____________________(1)  🞏 Not Documented(2) | 🞏 VA Pharmacy(1)  🞏 VA Clinic Stock(2)  🞏 Outside Pharmacy(3)  🞏 Other(4)  🞏 Unknown(5) |

**Outcomes:**

**28. Did the patient have a return urgent care/ED/primary care visit within 30 days of the index visit related to the urine index culture or urinary tract symptoms? (check only one box)**

**[Do not include other previously scheduled appointments (i.e., routine scheduled follow-up during index visit, orthopedic clinic, dermatology clinic, etc.)**

🞏 a. Yes 🞏 b. No

29. If yes to question #28, indicate where the return visit occurred (Select ONE the FIRST revisit):

🞏 a. Primary Care Clinic 🞏 b. Urgent Care Clinic 🞏 c. Emergency Dept 🞏 d. Specialty clinics: (Drop down for (1) Women’s, (2) Geri, (3) HBPC)

🞏 e. Unknown

**30 . If yes to question #28, which of the following conditions below best describe the reason for the return urgent care/ED/primary care visit? (check only one box)**

🞏 a. Patient had new/unresolved/worsening urinary tract symptoms

🞏 b. Patient had presumptive infection of unknown or other origin

🞏 c. Patient had symptoms consistent with an adverse antibiotic reaction (Select appropriate level of association from scroll down based on Liverpool causation tool (1. unlikely, 2. possible, 3. probable, 4. definite)

🞏 d. Patient had issues or questions regarding catheter placement/removal.

🞏 e. Other reason for return visit.

31. If yes to question 30c., please indicate which adverse antibiotic reaction symptom class was suspected from dropdown (can select more than 1) (a. Allergy/Immune, b. GI, c. skin, d. cardiac, e. hepatic, f. renal, g. pulmonary, h, acid-base/lytes, i. endocrine, j. neuro, k. other: __________________________)

**32 . If yes to question #28, did the patient have an antimicrobial prescribed as a result of the return visit ? (check only one box)**

🞏 1.Yes 🞏 2. No

# 2. Metric list for database (CDW) and chart review (EHR)

Primary Indicators

Provider-level proportions on key UTI-related measures obtained from chart review (EHR):

a. Proportion of ASB per urine culture with positive bacterial growth

b. Proportion of ASB treated with antibiotics

c. Proportion of acute simple cystitis treated with appropriate antibiotic selection

d. Proportion of acute simple cystitis treated with an appropriate duration of therapy

e. Proportion of acute simple cystitis with a fluoroquinolone

f. Proportion of ASB treated with a fluoroquinolone

Secondary Indicators

Provider-level rates on key UTI-related measures during the period of observation from data within the database (CDW):

a. Urinalyses ordered per 100 encounters

b. Urinalyses ordered with antibiotics prescribed per 100 encounters

c. Urine culture ordered per 100 encounters

d. Urine culture ordered per 100 urinalyses ordered

e. Urine culture ordered with antibiotics prescribed per 100 encounters

f. UTI diagnosis (excluding pyelonephritis) per 100 encounters

g. UTI diagnosis (excluding pyelonephritis) with antibiotics prescribed per 100 encounters

h. UTI diagnosis (excluding pyelonephritis) with antibiotics prescribed without urine culture ordered per 100 encounters

i. Proportion of UTI diagnoses (excluding pyelonephritis) treated with a fluoroquinolone

j. Proportion of UTI diagnoses (excluding pyelonephritis) with duration of antibiotics > 7 days

# 3. Preferred therapy selection and duration definitions

Acute Simple Cystitis (ASC) Level 1 Appropriate Selection Flag. Defined as initial therapy of a single drug. Trimethoprim-sulfamethoxazole (TMP/SMX), nitrofurantoin, or beta-lactams as initial therapy for the ASC cohort will be deemed Level 1 Appropriate therapy **unless** **any** of the following are present:

1) allergy to Level 1 antibiotics (TMP/SMX and nitrofurantoin -or- beta-lactams)

2) rationale documented in progress notes that contraindicate/preclude patient from receiving prescription of ALL TMP/SMX and nitrofurantoin and beta-lactams

(Note: We exclude “others” because can’t determine “appropriateness.” Fosfomycin excluded but captured in ASC counts.)

ASC Level 2 Appropriate Selection Flag. Defined as initial therapy of a single drug. Level 2 for ASC Is fluoroquinolones (cipro, levo) for **all the ASC cohort** which will be considered appropriate therapy ***only if***  patients have ***at least one*** of the following present:

1) allergy to both SMX/TMP and nitrofurantoin

2) allergy to aminopenicillins or cephalosporins

3) rationale documented in progress notes that contraindicate/preclude prescription of BOTH TMP/SMX and nitrofurantoin

4) rationale documented in progress notes that contraindicate/preclude prescription of beta-lactams

AND none of the following are present for Level 2:

1) allergy to fluoroquinolones

2) prior urine culture in past 3 months indicates resistance to fluoroquinolones

*NOTE: Only pulled prior urine culture data on select patients with current urine culture isolate as ESBL

ASC Appropriate Duration Flag. Defined via initial therapy of a single oral drug.

Appropriate duration of therapy for **acute simple cystitis** would be ***any*** of the following based on patient sex and initial antibiotic:

- 3 days for TMP/SMX for women
- 5 days for nitrofurantoin for women
- 7 days for β-lactam therapy for women
- 3 days for ciprofloxacin or levofloxacin for women
- 7 days for antibiotics for men (except levofloxacin as 5-days)

ASC Appropriate Selection AND Duration. This flag would be selected if the patient met at least one level appropriate selection for ASC AND appropriate duration for ASC.

Uncomplicated pyelonephritis (UPN) Level 1 Appropriate Selection Flag. Defined as initial therapy of a single drug. TMP/SMX, ciprofloxacin, or levofloxacin is appropriate unless there is at least one multidrug resistance (MDR) factor, or 3 month history of resistance, allergy, or contraindications to BOTH types of drug (TMP/SMX and fluoroquinolones). Single dose of ceftriaxone is acceptable prior to these antibiotics.

UPN Level 2 Appropriate Selection Flag. Defined as initial therapy of a single drug.

If there is at least ONE MDR risk factor (Q6 from InfoPath), then a single dose of carbapenem/aminoglycoside is appropriate followed by an oral therapy including ciprofloxacin, levofloxacin, TMP/SMX, advanced generation cephalosporins, or amoxicillin/clavulanate as long as the patient **does not** have a 3 month hx of resistance against the chosen drug, allergy, or contraindications to the CHOSEN drug.

UPN Appropriate Duration Flag.

Appropriate duration of therapy for **uncomplicated pyelonephritis** would be ***any*** of the following based on initial antibiotic:

- 14 days TMP/SMX
- 5-7 days fluoroquinolone
- 10-14 days’ supply for beta-lactams
- All other drugs would be >7 days’ supply

UPN Appropriate Selection AND Duration Flag. This flag would be selected if the patient met at least one level appropriate selection for UPN AND appropriate duration for UPN.

# 4. Urinary Isolate Breakdown

**Figure 4-1. Urinary Isolate Breakdown.**


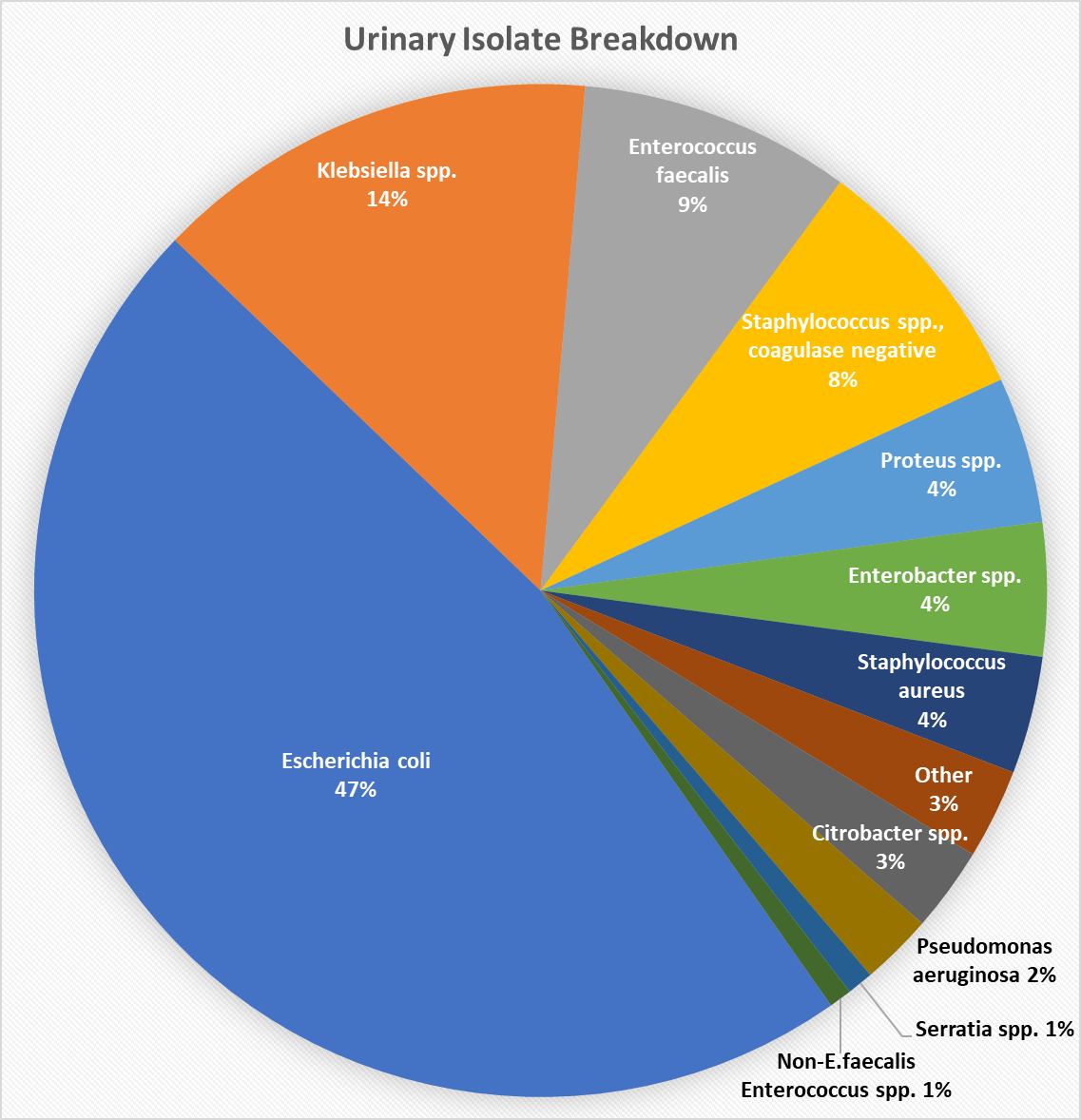


# 5. Pathogen sensitivities

**Table 5-1. Pathogen sensitivities.**

|  | Amox/  Clav^A^ | Amox/  clav, susc % | Cef-azolin^A^ | Cefazolin, susc. % | Cef-triaxone^A^ | Cef-triaxone, susc. % | Cipro-floxacin + Levo-floxacin^A^ | Cipro-floxacin + Levo-floxacin, susc. % | Erta-penem^A^ | Erta-penem, susc. % | Genta-micin^A^ | Genta-micin, susc. % | Nitro-furantoin^A^ | Nitro-furantoin, susc. % | TMP/  SMX^A^ | TMP/  SMX, susc. % |
| --- | --- | --- | --- | --- | --- | --- | --- | --- | --- | --- | --- | --- | --- | --- | --- | --- |
| *Escherichia coli* | 342  416 | 82 | 921  1052 | 88 | 1073  1146 | 94 | 1021  1282 | 80 | 681  682 | 100 | 1209  1283 | 94 | 1189  1236 | 96 | 1032  1283 | 80 |
| *Klebsiella spp.* | 112  123 | 91 | 284  329 | 86 | 336  356 | 94 | 376  394 | 95 | 216  217 | 100 | 383  391 | 98 | 201  381 | 53 | 358  391 | 92 |

Legend: A- Denominator in column indicates number of pathogens in cohort with reported sensitivities for that antibiotic; numerator indicates number of pathogens sensitive to that antibiotic; these numbers were from the initial data pull from both included and excluded patients

# 6. Additional outcome analyses

**Table 6-1. Clinical Outcomes for Patient Cases with Asymptomatic Bacteriuria**

| **Antibiotic** | **Urinary-related Return Visit within 30 days** | **Clinical Failure within 30 days^A^** | **All-cause Hospitalizations within 30 days** | | **Composite of 30-day clinical failure and all-cause hospitalizations** | | | |
| --- | --- | --- | --- | --- | --- | --- | --- | --- |
|  | **n (%)** | **n (%)** | **n (%)** | **OR** | | | **+ 95% CI** | **p-Value** |
| Without antibiotics (n=983) | 66 (7) | 43 (4) | 13 (1) | Reference | |  | |  |
| With antibiotics (n=628) | 70 (11) | 45 (7) | 29 (5) | 2.21 | | 1.51, 3.24 | | <0.001 |

**Legend:** A- Clinical failure is defined as a return visit with new, unresolved, or worsening UTI symptoms; 1% of asymptomatic bacteriuria cases were removed from this subgroup due to (1) indeterminant definitions for appropriate selection and (3) data missingness

**Figure 6-1. Composite clinical failure rates among beta-lactams in cystitis and pyelonephritis cases.**

**Table 6-2. Clinical Outcomes for Patient Cases with Acute Cystitis and Pyelonephritis by Preferred Antibiotic Duration**

|  | **Urinary-related Return Visit within 30 days** | **Clinical Failure within 30 days^A^** | **All-cause Hospitalizations within 30 days** | | **Composite of 30-day clinical failure and all-cause hospitalizations** | | | |
| --- | --- | --- | --- | --- | --- | --- | --- | --- |
|  | **n (%)** | **n (%)** | **n (%)** | **OR** | | | **+ 95% CI** | **p-Value** |
| Preferred duration (n=511) | 100 (20) | 74 (14) | 23 (5) | Reference | |  | |  |
| Shorter duration (n=199) | 39 (20) | 27 (14) | 19 (10) | 1.27 | | 0.84, 1.92 | | 0.253 |
| Longer duration  (n=510) | 77 (15) | 50 (10) | 18 (4) | 0.66 | | 0.46, 0.93 | | **0.017** |

**Legend:** A- Clinical failure is defined as a return visit with new, unresolved, or worsening UTI symptoms; 23% of cystitis cases were removed from this subgroup due to (1) indeterminant definitions for appropriate selection and/or duration (*n*=26); (2) no antibiotics were received (*n*=198); (3) data missingness (i.e. antibiotic name or duration was not documented by chart reviewer) (*n*=38); 31% of pyelonephritis cases were removed from this subgroup due to (1) indeterminant definitions for appropriate selection and/or duration (*n*=79); (2) no antibiotics were received (*n*=46); (3) data missingness (i.e. antibiotic name or duration was not documented by chart reviewer) (*n*=19)

**Table 6-3. Beta-Lactams: Clinical Outcomes for Patient Cases with Acute Cystitis and Pyelonephritis by Preferred Antibiotic Duration**

|  | **Urinary-related Return Visit within 30 days** | **Clinical Failure within 30 days^A^** | **All-cause Hospitalizations within 30 days** | | **Composite of 30-day clinical failure and all-cause hospitalizations** | | | |
| --- | --- | --- | --- | --- | --- | --- | --- | --- |
|  | **n (%)** | **n (%)** | **n (%)** | **OR** | | | **+ 95% CI** | **p-Value** |
| Preferred duration (n=134) | 30 (22) | 20 (15) | 10 (7) | Reference | |  | |  |
| Shorter duration (n=58) | 12 (21) | 9 (16) | 6 (10) | 1.21 | | 0.55, 2.59 | | 0.585 |
| Longer duration  (n=82) | 19 (23) | 13 (16) | 4 (5) | 0.91 | | 0.43, 1.86 | | 0.866 |

**Legend:** A- Clinical failure is defined as a return visit with new, unresolved, or worsening UTI symptoms; 23% of cystitis cases were removed from this subgroup due to (1) indeterminant definitions for appropriate selection and/or duration (*n*=26); (2) no antibiotics were received (*n*=198); (3) data missingness (i.e. antibiotic name or duration was not documented by chart reviewer) (*n*=38); 31% of pyelonephritis cases were removed from this subgroup due to (1) indeterminant definitions for appropriate selection and/or duration (*n*=79); (2) no antibiotics were received (*n*=46); (3) data missingness (i.e. antibiotic name or duration was not documented by chart reviewer) (*n*=19)

**Table 6-4. Fluoroquinolones: Clinical Outcomes for Patient Cases with Acute Cystitis and Pyelonephritis by Preferred Antibiotic Duration**

|  | **Urinary-related Return Visit within 30 days** | **Clinical Failure within 30 days^A^** | **All-cause Hospitalizations within 30 days** | | **Composite of 30-day clinical failure and all-cause hospitalizations** | | | |
| --- | --- | --- | --- | --- | --- | --- | --- | --- |
|  | **n (%)** | **n (%)** | **n (%)** | **OR** | | | **+ 95% CI** | **p-Value** |
| Preferred duration (n=209) | 38 (18) | 30 (14) | 7 (3) | Reference | |  | |  |
| Shorter duration (n=41) | 6 (15) | 5 (12) | 3 (7) | 1.09 | | 0.40, 2.67 | | 0.827 |
| Longer duration  (n=210) | 25 (12) | 15 (7) | 4 (2) | 0.46 | | 0.24, 0.86 | | **0.010** |

**Legend:** A- Clinical failure is defined as a return visit with new, unresolved, or worsening UTI symptoms

**Table 6-5. TMP-SMX: Clinical Outcomes for Patient Cases with Acute Cystitis and Pyelonephritis by Preferred Antibiotic Duration**

|  | **Urinary-related Return Visit within 30 days** | **Clinical Failure within 30 days^A^** | **All-cause Hospitalizations within 30 days** | | **Composite of 30-day clinical failure and all-cause hospitalizations** | | | |
| --- | --- | --- | --- | --- | --- | --- | --- | --- |
|  | **n (%)** | **n (%)** | **n (%)** | **OR** | | | **+ 95% CI** | **p-Value** |
| Preferred duration (n=81) | 18 (22) | 12 (15) | 3 (4) | Reference | |  | |  |
| Shorter duration (n=82) | 16 (20) | 11 (13) | 7 (9) | 1.23 | | 0.54, 2.88 | | 0.697 |
| Longer duration  (n=127) | 16 (13) | 11 (9) | 6 (5) | 0.68 | | 0.30, 1.57 | | 0.331 |

**Legend:** A- Clinical failure is defined as a return visit with new, unresolved, or worsening UTI symptoms

# 7. Adverse Drug Event Outcomes

**Table 7-1. Adverse Drug Event Outcomes by Preferred and Non-preferred Therapy.**

| *Expressed as per 1000 urine-cultures | Preferred Therapy  (n=1775) | Non-preferred Therapy (n=1436) |
| --- | --- | --- |
| Patient had an ADR (n=16) | 5.1 | 4.9 |
| Possible ADR (n=7) | 2.3 | 2.1 |
| Probable ADR (n=2) | 0.6 | 0.7 |
| Definitive ADR (n=3) | 1.1 | 0.7 |
| Allergy/Immune | 1.1 | 0.0 |
| GI | 1.7 | 2.1 |
| Skin or structure | 1.1 | 0.0 |
| Neuro | 0.6 | 0.7 |
| Other | 0.6 | 1.4 |
| Other comments | - swollen extremities & abdominal discomfort | - Hematological - warfarin interaction-> high INR |

**Table 7-2. Adverse Drug Event Outcomes by Preferred and Non-preferred ASB Therapy.**

| *Expressed as per 1000 urine-cultures | Preferred ASB Therapy  (n=983) | Non-preferred ASB Therapy (n=628) |
| --- | --- | --- |
| Patient had an ADR (n=8) | 3.1 | 8.0 |
| Possible ADR (n=3) | 1.0 | 3.2 |
| Probable ADR (n=2) | 1.0 | 1.6 |
| Definitive ADR (n=0) | 0.0 | 0.0 |
| Allergy/Immune (n=2) | 2.0 | 0.0 |
| GI (n=3) | 1.0 | 3.2 |
| Other (n=2) | 0.0 | 3.2 |
| Other comments | N/A | - Hematological - warfarin interaction-> high INR |

**Table 7-3. Adverse Drug Event Outcomes by Different Drugs and Drug Classes.**

|  | GI (n=5) | Skin (n=3) | Neuro (n=2) | Other (n=4) |
| --- | --- | --- | --- | --- |
| Amoxicillin (n=43) | 0 | 0 | 0 | 0 |
| Amoxicillin/clavulanate (n=112) | 1 | 0 | 0 | 0 |
| 1st gen Cephalosporin (n=127) | 0 | 0 | 0 | 0 |
| Advanced generation Cephalosporin (n=136) | 0 | 0 | 1 | 0 |
| Fluoroquinolones (n=677) | 2 | 2 | 0 | 2 |
| TMP/SMX (n=448) | 2 | 1 | 0 | 1 |
| Nitrofurantoin (n=411) | 0 | 0 | 1 | 1 |
| Tetracycline (n=39) | 0 | 0 | 0 | 0 |

# 8. Linear regression Analyses of Provider-level Database-derived and Chart-derived Measure of UTI Management.

| Independent Variable | Dependent Variable | | Slope equation | Coefficient of determination (R^2^) | | p-value |
| --- | --- | --- | --- | --- | --- | --- |
| UAs ordered with antibiotics prescribed within 4-day time window (+/- 2 days) per 100 encounters | Proportion of ASB per urine culture with positive bacterial growth | y = -20.9x + 0.6 | | 0.38 | <0.001 | |
| Urine cultures ordered per 100 encounters | Proportion of ASB per urine culture with positive bacterial growth | y = -0.6x + 0.6 | | 0.06 | 0.011 | |
| Urine cultures ordered per 100 urinalyses ordered | Proportion of ASB per urine culture with positive bacterial growth | y = -0.3x + 0.6 | | 0.13 | <0.001 | |
| UCs ordered with antibiotics prescribed within 4-day time window (+/- 2 days) per 100 encounters | Proportion of ASB per urine culture with positive bacterial growth | y = -21.7x + 0.6 | | 0.40 | <0.001 | |
| UTI diagnoses (excluding pyelonephritis) per 100 encounters | Proportion of ASB per urine culture with positive bacterial growth | y = -21.4x + 0.4 | | 0.38 | <0.001 | |
| UTI diagnosis (excluding pyelonephritis) with antibiotics prescribed within 4-day time window (+/- 2 days) per 100 encounters | Proportion of ASB per urine culture with positive bacterial growth | y = -20.1x + 0.6 | | 0.35 | <0.001 | |
| Urinalyses ordered with antibiotics prescribed within 4-day time window (+/- 2 days) per 100 encounters | Proportion of ASB treated with antibiotics | y = 23.4x + 0.4 | | 0.30 | <0.001 | |
| Urine culture ordered per 100 urinalyses ordered | Proportion of ASB treated with antibiotics | y = 0.3x+0.1 | | 0.10 | 0.001 | |
| UCs ordered with antibiotics prescribed within 4-day time window (+/- 2 days) per 100 encounters | Proportion of ASB treated with antibiotics | y = 24.1x+0.3 | | 0.31 | <0.001 | |
| UTI diagnosis (excluding pyelonephritis) per 100 encounters | Proportion of ASB treated with antibiotics | y = 22.9x+0.3 | | 0.27 | <0.001 | |
| UTI diagnosis (excluding pyelonephritis) with antibiotics prescribed within 4-day time window (+/- 2 days) per 100 encounters | Proportion of ASB treated with antibiotics | y = 22.4x+0.3 | | 0.27 | <0.001 | |
| Proportion of UTI diagnoses (excluding pyelonephritis) treated with a fluoroquinolone | Proportion of acute simple cystitis treated with appropriate antibiotic selection | y = -0.6x+0.8 | | 0.24 | <0.001 | |
| Proportion of UTI diagnoses (excluding pyelonephritis) with duration of antibiotics > 7 days | Proportion of acute simple cystitis treated with an appropriate duration of therapy | y =-0.3x +0.5 | | 0.11 | <0.001 | |
| Proportion of UTI diagnoses (excluding pyelonephritis) treated with a fluoroquinolone | Proportion of acute simple cystitis with a fluoroquinolone | y = 0.6x + 0.1 | | 0.27 | <0.001 | |

**Legend:** UTI, Urinary tract infection; ASB, asymptomatic bacteriuria; UC, urine culture; UAs, urinalyses
